# The role of SK3 in progesterone-induced inhibition of human fallopian tubal contraction

**DOI:** 10.1186/s12958-022-00932-3

**Published:** 2022-04-29

**Authors:** Duo Zhang, Qian Zhu, Wei Xia, Chenfeng Zhu, Xiaoya Zhao, Yiqin Zhang, Chuqing He, Sifan Ji, Xiaocui Li, Jian Zhang

**Affiliations:** 1 Department of Obstetrics and Gynecology, International Peace Maternity and Child Health Hospital, School of Medicine, Shanghai Jiaotong University, Shanghai, 200030 China; 2 Shanghai Key Laboratory of Embryo Original Diseases, Shanghai, 200030 China; 3 Department of Obstetrics and Gynecology, Shanghai First Maternity and Infant Hospital, School of Medicine, Tongji University, Shanghai, 200092 China

**Keywords:** Progesterone, Levonorgestrel, Tubal pregnancy, Ectopic pregnancy, SK3, Fallopian tube, Smooth muscle

## Abstract

**Background:**

Normal motor activity of the fallopian tube is critical for human reproduction, and abnormal tubal activity may lead to ectopic pregnancy (EP) or infertility. Progesterone has an inhibitory effect on tubal contraction; however, the underlying mechanisms remain unclear. Small-conductance calcium-activated K^+^ channel 3 (SK3) is abundantly expressed in platelet-derived growth factor receptor α positive (PDGFRα+) cells and was reported to be important for the relaxation of smooth muscle. The present study aims to explore the expression of SK3 in the human fallopian tube and its role in progesterone-induced inhibition of tubal contraction.

**Methods:**

We collected specimens of fallopian tubes from patients treated by salpingectomy for EP (EP group) and other benign gynecological diseases (Non-EP group). The expression of SK3 was detected by quantitative real-time polymerase chain reaction, western blot, immunocytochemistry, and immunohistochemistry analyses. Isometric tension experiments were performed to investigate the role of SK3 in progesterone-induced inhibition of tubal contraction.

**Results:**

The baseline amplitude and frequency of human fallopian tube contraction were both statistically lower in the EP group compared with the non-EP group. The expression levels of SK3 in different portions of fallopian tubes from the non-EP group were significantly higher than in those from the EP group. Progesterone had an inhibitory effect on tubal contraction, mainly on the amplitude, in both groups, and SK3 as well as other calcium-activated K^+^ channels may be involved. SK3-expressing PDGFRα (+) cells were detected in the human fallopian tube.

**Conclusions:**

The expression of SK3 is lower in the EP group, and SK3 is involved in the progesterone-induced inhibition of human fallopian tube contraction.

**Supplementary Information:**

The online version contains supplementary material available at 10.1186/s12958-022-00932-3.

## Background

After the advent of in vitro fertilization and embryo transfer, studies of the function of the fallopian tube were neglected. In fact, the fallopian tube is not only a muscular pathway for gametes or embryos, but is also involved in many other physiological processes, including the storage and release of sperm, regulation of fertilization, and identification of unfertilized eggs and early embryos [1]. Tubal cilia movements, smooth muscle contraction, and tubal secretory fluids play important roles in tubal transportation, which is regulated by complex mechanisms [2, 3]. Normal tubal function is critical for reproduction, and structural and functional abnormalities in the fallopian tube may result in ectopic pregnancy (EP) or tubal-related infertility [2–4].

EP is defined as the implantation of the embryo outside the uterine cavity and has a prevalence of about 1.5–2%; more than 95% of EPs occur in the fallopian tube [3, 5]. Because of the damage caused by EP lesions or subsequent surgery, EP is a major cause of maternal hemorrhage-related mortality in the first trimester and may also be a risk factor for future infertility and recurrent EP [5]. As revealed in a previous study, the risk of EP is significantly higher following the failure of emergency contraception (EC) with levonorgestrel (LNG, a type of progestogen) [6]. Furthermore, in studies aimed at explored the underlying mechanisms it was found that both progesterone and LNG rapidly decrease the cilia beat frequency in a concentration-dependent manner [7, 8]. Wanggren K et al. demonstrated that the application of progesterone or LNG rapidly inhibits human fallopian tubal contraction; however, the mechanisms remain unknown [9].

Progesterone leads to myometrial relaxation through genomic and nongenomic pathways [10]. It is believed that most of progesterone-induced effects are mediated by the specific binding between progesterone and its nuclear receptor with subsequent change in expression of target gene [10]. Specifically, progesterone inhibits the expression of connexin 43, estradiol-induced increase in cGMP-dependent protein kinase and the expression level of interleukin-8 in myometrium stromal cells [10]. The nongenomic mechanism, which includes inhibition of transmembrane Ca^2+^ flow, intracellular Ca^2+^ release, and cell hyperpolarization, seems to be a more convincing explanation for the rapid effects of progesterone [10, 11].

Calcium-activated K^+^ channels (KCas) are an important subfamily of K^+^ channels that share a common function—coupling an increase in intracellular Ca^2+^ concentration to cell hyperpolarization [12, 13]. KCas can be divided into three types based on conductance: big (BKCa), intermediate (IKCa) and small (SKCa) conductance calcium-activated K^+^ channels [12, 13]. It was reported that SK3, the most widely expressed subtype of SKCas in humans, is involved in many biological functions including cell migration and invasion, blastocyst hatching, and inflammatory responses, as well as many pathophysiological processes including atrial fibrillation, detrusor overactivity, neuroendocrine differentiation, and drug resistance in cancer [13–18]. Notably, SK3 is important in the contraction of skeletal muscle and smooth muscle in the gastrointestinal tract, uterus, and bladder [19–22]. The expression of SK3 in the human myometrium was demonstrated by Mazzone et al. early in 2002, and the inhibitory effect of SK3 in uterine contraction was observed in rodents [23–25]. With respect to the potential mechanisms, platelet-derived growth factor receptor α positive (PDGFRα+) cells, a class of interstitial cells with abundant expression of SK3, also previously known as fibroblast-like cells, may play a significant role in attenuating the excitability and contractility of muscle [14, 26, 27]. In 2015, Peri LE et al. found that PDGFRα (+) cells exist in reproductive organs including the ovary, uterus, and fallopian tube from mice and monkeys [28]. However, the expression of SK3 and the presence of PDGFRα (+) cells in human fallopian tube have not been investigated.

The present study aimed to detect the expression of SK3 in human fallopian tube smooth muscle (hFTSM) from patients with EP and to investigate its potential function in the progesterone-mediated inhibition of tubal contraction. Furthermore, we explored the existence of SK3-expressing PDGFRα (+) cells in hFTSM.

## Methods

### Ethical statement

The study was approved by the Ethics Committee of the International Peace Maternity and Child Health Hospital ([GKLW]2018–04). Patients were assured that their information would be kept strictly confidential and that the study posed no short- or long-term potential risks to them. Written informed consent was obtained from every patient before the operation. All specimens were collected according to the guidelines of the Declaration of Helsinki.

### Sample collection

Patients who underwent total hysterectomy combined with bilateral salpingectomy for uterine fibroids (proliferative stage) (Non-EP group) and patients who underwent salpingectomy for EP (EP group) were included from the International Peace Maternal and Child Health Hospital in Shanghai, China.

Specimens from the interstitial portion of the fallopian tube, isthmus, ampulla, and infundibulum were obtained from patients undergoing hysterectomy and salpingectomy. However, for women with pathologically confirmed tubal pregnancy, it was difficult to obtain the interstitial portion of the fallopian tube because it passes through the uterine muscular layer. Each specimen was divided into at least three parts: one part was left for pathological examination; one part was fixed in 4% paraformaldehyde (PFA, Wuhan Servicebio Technology Co., Ltd., Wuhan, China) for immunohistochemistry and immunofluorescence analyses; and one part was immediately frozen in liquid nitrogen for reverse-transcription polymerase chain reaction (RT-PCR) and western blot analyses. For the ampulla smooth muscle, an additional portion of the specimen was kept in ice-cold Krebs solution for isometric tension experiment (only the smooth muscle from the ampulla was large enough for isometric tension experiments).

### RNA extraction and real-time quantitative RT-PCR (qRT-PCR)

Total RNA was extracted from hFTSM tissue using TRIzol reagent (Roche Applied Science, Penzberg, Germany). The quality and quantity of RNA were examined using a NanoDrop 2000/2000c Spectrophotometer (Thermo Fisher Scientific Inc., Massachusetts, USA). The mRNA was converted to cDNA using the PrimerScript™ RT reagent Kit with gDNA Eraser (Takara, Dalian, China; cat. no. RR047). Two-step real-time PCR was performed on a QuantStudio 7 Flex PCR instrument (Applied Biosystems, Foster City, CA, USA) using SYBR Premix Ex Taq (Takara; cat. no. RR420) according to the manufacturer’s protocol under the following cycling conditions: 95 °C for 30 s, and 60 °C for 31 s, and 72 °C for 30 s for 40 cycles. The specific primer sequences used in this study were: 5′-CACATGGCCTCCAAGGAGTAA-3′ (forward) and 5′-TGAGGGTCTCTCTCTTCCTCTTGT-3′ (reverse) for GAPDH and 5′-TGGGAAAGGTGTGTCTGTCTCC-3′ (forward) and 5′-CTTGGTGAGCTGAGTGTCCA-3′ (reverse) for SK3. The primers were designed and synthesized by Sangon Biotech (Shanghai, China). Reactions were carried out in three replicates for each sample.

### Immunohistochemistry

After removal of mesosalpinx and adipose tissues covering the tubal serosa, the tissue was fixed in formalin, embedded in paraffin, and cut into 5 μm sections. The sections were then deparaffinized and rehydrated. After incubation in 3% H_2_O_2_ to block endogenous peroxidases, the sections were treated with heated antigen retrieval reagent containing EDTA (Servicebio Technology). The sections were incubated with protein-block serum-free reagent (Servicebio Technology) for 30 min to reduce non-specific binding and then incubated with a primary antibody for SK3 at 4 °C overnight, followed by incubation with a secondary antibody for 30 min at room temperature. Isotype control immunoglobulin (rabbit IgG) was used as a substitute for the primary antibody. Stained slides were photographed under a light microscope (Leica, Wetzlar, Germany) at 100× and 200× magnification.

Smooth muscle tissue from the tubal ampulla were fixed in 4% PFA, dehydrated in 20–30% sucrose solutions, and embedded in optimal cutting temperature compound. Next 5 μm sections were cut and counted. The sections were blocked for 30 min at room temperature and incubated with primary antibodies for α-SMA or PDGFRα at 4 °C overnight. Then the sections were incubated with secondary antibodies and 4′,6-diamidino-2-phenylindole (DAPI) (1:500; Invitrogen, Carlsbad, CA, USA). Images were captured by fluorescence microscopy. All antibodies used for immunohistochemistry are listed in S Table 1.

### Western blotting

HFTSM tissue was washed with phosphate buffered saline (PBS) (Sangon Biotech) and lysed with 250 μl radioimmunoprecipitation assay lysis buffer (Beyotime Institute of Biotechnology, Hangzhou, China; cat.no. P0013B) containing PMSF (1:100) on ice for 30 min. Next, the homogenate was centrifuged at 12,000 rpm at 4 °C for 10 min and the supernatant was collected. The protein concentration of the supernatant was determined by bicinchoninic acid (BCA) assay using a BCA Protein Assay Kit (Beyotime). Equivalent amounts of protein were loaded and separated on a 10% sodium dodecyl sulfate-polyacrylamide gel and then transferred to polyvinylidene fluoride membranes (Millipore, Billerica, MA, USA). The membranes were blocked with bovine serum albumin (BSA) (Gibco, Rockville, MD, USA) for 1 h and incubated with primary antibodies (listed in S Table 1) at 4 °C overnight. After washing three times with tris buffered saline containing Tween-20 (0.05%), horseradish peroxidase-labeled anti-rabbit IgG (details in S Table 1) was used as the secondary antibody for the detection of the primary antibody. The signals were measured using an enhanced chemiluminescence detection kit (Yeasen Biotechnology Co., Ltd. Shanghai, China; cat. no. 36208ES60).

### Primary cultures of hFTSM cells

After the removal of the tubal serosa and endometrium, smooth muscle tissue was minced into small pieces in Hanks’ Balanced Salt Solution (Byotime, cat. no. C0218) and digested with collagenase II (Solarbio Science & Technology Co., Ltd., Beijing, China. cat. no. C8150) and DNase I (Solarbio; cat. no. D8071) for 1 h at 37 °C followed by addition of fetal bovine serum (FBS; Gibco, Rockville, MD, USA). After digestion, smooth muscle cells (SMCs) were filtered using a 10 μm nylon cell strainer and centrifuged at 1000 rpm for 5 min. The pelleted cells were re-suspended with Dulbecco’s Modified Eagle’s Medium/F12 (Gibco) containing 10% FBS and penicillin/streptomycin (Gibco), seeded into a 6-well plate, and cultured at 37 °C in a humidified atmosphere containing 5% CO_2_.

### Immunocytochemistry

Cultured cells were rinsed with PBS and then fixed with 4% PFA for 20 min. Cells were treated for 5–10 min with 0.5% Triton-100 (Beyotime; cat. no. P0096) and then 4% BSA was added to reduce non-specific staining. Specimens were incubated with primary antibodies (S Table 1) at 37 °C overnight, followed by incubation with secondary antibodies (S Table 1). For the negative controls, the primary antibodies were replaced with PBS and cells were only incubated with the secondary antibodies. The specimens were analyzed and photographed using a Zeiss LSM 780 confocal microscope (Zeiss, Jena, Germany).

### Preparation of the hFTSM and isometric tension measurement

Human fallopian tube tissue was collected and placed in a pre-oxygenated (95% O2:5% CO2) Krebs solution at 4 °C. The components of the Krebs solution are listed in S Table 2. The fallopian tube was cut open along the longitudinal axis and was rinsed repeatedly with Krebs solution to remove the blood and mucus. The mesosalpinx and adipose tissues covering the serosal surface of the fallopian tube were carefully removed. Then the tissue was unfolded and pinned with the mucosa facing upwards to a Sylgard base dish filled with Krebs solution continuously bubbled with 95% O_2_ and 5% CO_2_. The mucosa and submucosa were removed carefully under a dissecting microscope (Olympus Optical Co., Ltd. Tokyo, Japan), and smooth muscle strips (2 mm × 8 mm) were obtained by cutting along the circular axis of the tubal cavity. Both ends of the smooth muscle strip were tied with a piece of silk thread (USP 5/0), and the smooth muscle strip was placed along the longitudinal axis of a 10 ml organ bath containing 37 °C Krebs solution bubbled with 95% O_2_ and 5% CO_2_. The mechanical activity was examined and recorded using an isometric force transducer (RM6240C; Chengdu Instrument Factory, Sichuan, China) linked to an amplifier device. A tension of 0.3 g was applied to the smooth muscle strip, and the strip was re-equilibrated for 30–40 min for recovery of contraction. To distinguish SK3 from BKCa and IKCa, hFTSM strips were incubated with apamin (300 nM), a widely used specific blocker of SK3, and tetraethylammonium (TEA, 5 mM), a non-specific potassium channel blocker, before the addition of progesterone to the organ bath [12, 13, 29].

### Drugs and chemicals

Information about the drugs and chemicals used in the isometric tension measurements is shown in S Table 2.

### Data analysis and statistics

Data in figures are shown as the mean ± standard error of mean and data in Table 1 are shown in mean ± standard deviation. The relative expression of the genes of interest was normalized to GAPDH and quantified using the 2^△CT^ method. The relative amplitude of contraction was calculated by normalizing the amplitude of contraction to the baseline. Comparison of expression between different tubal sites within a group was performed by paired t-test. The Mann-Whitney U test was used to detect the differences in expression between the Non-EP and EP group. Spearman’s correlation test was used to evaluate the correlation between increasing concentrations of progesterone and amplitude. The intensities of immunoreactive bands were analyzed with Image J software (version 1.53, NIH, Bethesda, MD, USA). The isometric tension measurement data were analyzed and annotated by Igor Pro (version 2018, WaveMetrics, Portland, OR, USA) and CorelDRAW X7 (Corel Corporation, Ottawa, Canada). IBM SPSS Statistics (version 23.0, IBM Inc., Armonk, NY, USA) and GraphPad Prism (version 8, GraphPad Software, La Jolla, CA, USA) were also used for data analysis. *P* values less than 0.05 were considered statistically significant.

**Table 1:** Clinical characteristics of patients in Non-EP and EP group

	EP group (*n* = 20)	Non-EP group (*n* = 20)	*P* value
Age (year) ^a^	32.08 ± 5.28	48 ± 3.63	< 10^−4^
Weight (kg) ^a^	61.38 ± 10.56	57.03 ± 8.62	0.16
Gravidity ^a^	1.45 ± 1.19	2.40 ± 1.14	0.01
Parity ^a^	0.55 ± 0.60	1.05 ± 0.51	0.01
History of CS ^b^	3 (15%)	7 (35%)	0.14
History of EP ^b^	7 (35%)	0 (0%)	0.01

*EP* ectopic pregnancy, *CS* cesarean section

^a^ Data were shown in average ± standard deviation

^b^ Data were shown in number (proportion)

## Results

### Clinical characteristics of patients

From August 2018 to March 2019, a total of 40 patients were recruited to the Non-EP group (*n* = 20) and EP group (*n* = 20). The clinical characteristics of all recruited patients were shown in Table 1. The mean ages of patients in the Non-EP group and EP group were 48 ± 3.63 and 32.08 ± 5.28 years, respectively (*P* < 10^− 4^). The gravidity (*P* = 0.01) and parity (*P* = 0.01) in EP group were also significantly lower than Non-EP group. Patients with a history of EP, a well-known risk factor for EP, is markedly more in EP group than Non-EP group. However, no significant difference was observed in weight and history of cesarean section between two groups.

### The effect of progesterone on spontaneous contraction of the hFTSM

**Fig. 1:**
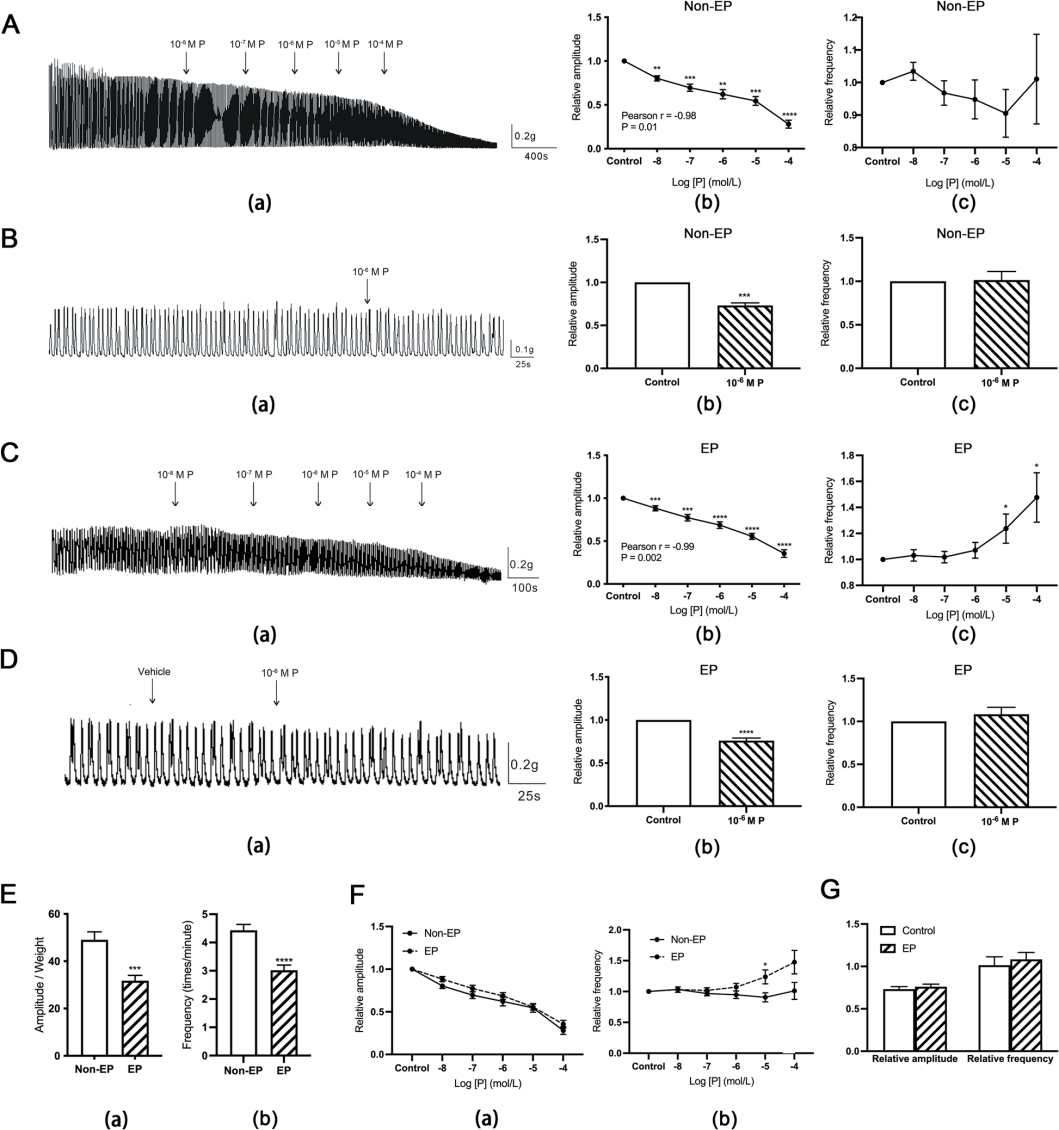
Effect of progesterone on spontaneous contraction of the hFTSM. **A**-**B** The effect of increasing concentrations of progesterone (**A**) and 10^− 6^ M progesterone (**B**) on the amplitude (**b**) and frequency (**c**) of spontaneous contraction of the hFTSM in the Non-EP group. **C**-**D** The effect of increasing concentrations of progesterone (**C**) and 10^− 6^ M progesterone (**D**) on the amplitude (**b**) and frequency (**c**) of spontaneous contraction of the hFTSM in the EP group. **E** The difference in baseline contraction amplitude (**a**) and frequency (**b**) between the Non-EP and EP groups. **F** The difference in the effect of increasing concentrations of progesterone on the amplitude (**a**) and frequency (**b**) of spontaneous contraction of the hFTSM between the two groups. **G** The difference in the effect of 10^− 6^ M progesterone on spontaneous contraction of the hFTSM between the two groups. *, *P*<0.05; **, *P*<0.01; ***, *P*<0.001; ****, *P*<0.0001

To detect the effect of progesterone on human fallopian tube contraction, we performed an isometric tension experiment in which the effects of increasing concentrations (10^− 8^–10^− 4^ M) of progesterone on contraction in the EP and Non-EP groups were compared. In both groups the amplitude decreased with an increase in progesterone concentration (Non-EP group: Fig. 1A; (b) Pearson R = -0.98, *P* = 0.01; EP group: Fig. 1C; (b) Pearson R = -0.99, *P* = 0.002). The amplitude was significantly reduced after the addition of 10^− 6^ M progesterone (Non-EP group: Fig. 1B (b), EP group: Fig. 1D (b)). The frequency of contraction remained the same as the baseline in the Non-EP and EP groups (Fig. 1A (c); Fig. 1B (c); Fig. 1D (c)). Interestingly, the frequency increased significantly in the EP group when a series of increasing concentrations of progesterone, ranging from 10^− 5^–10^− 4^ M, were added (Fig. 1C (c)). The effect of increasing concentrations of progesterone (Fig. 1F (a)) and 10^− 6^ M progesterone (Fig. 1G) on the contractile pattern were basically same in both groups, except that an elevated frequency was observed in EP group under 10^− 5^ M progesterone (Fig. 1F (b)). To conclude, increasing concentration of progesterone and 10^− 6^ M progesterone can both reduce the contractile amplitude of fallopian tube in two groups, and the frequency in EP group increased markedly under the effect of 10^− 5^–10^− 4^ M progesterone.

### The expression of SK3 in the hFTSM

**Fig. 2:**
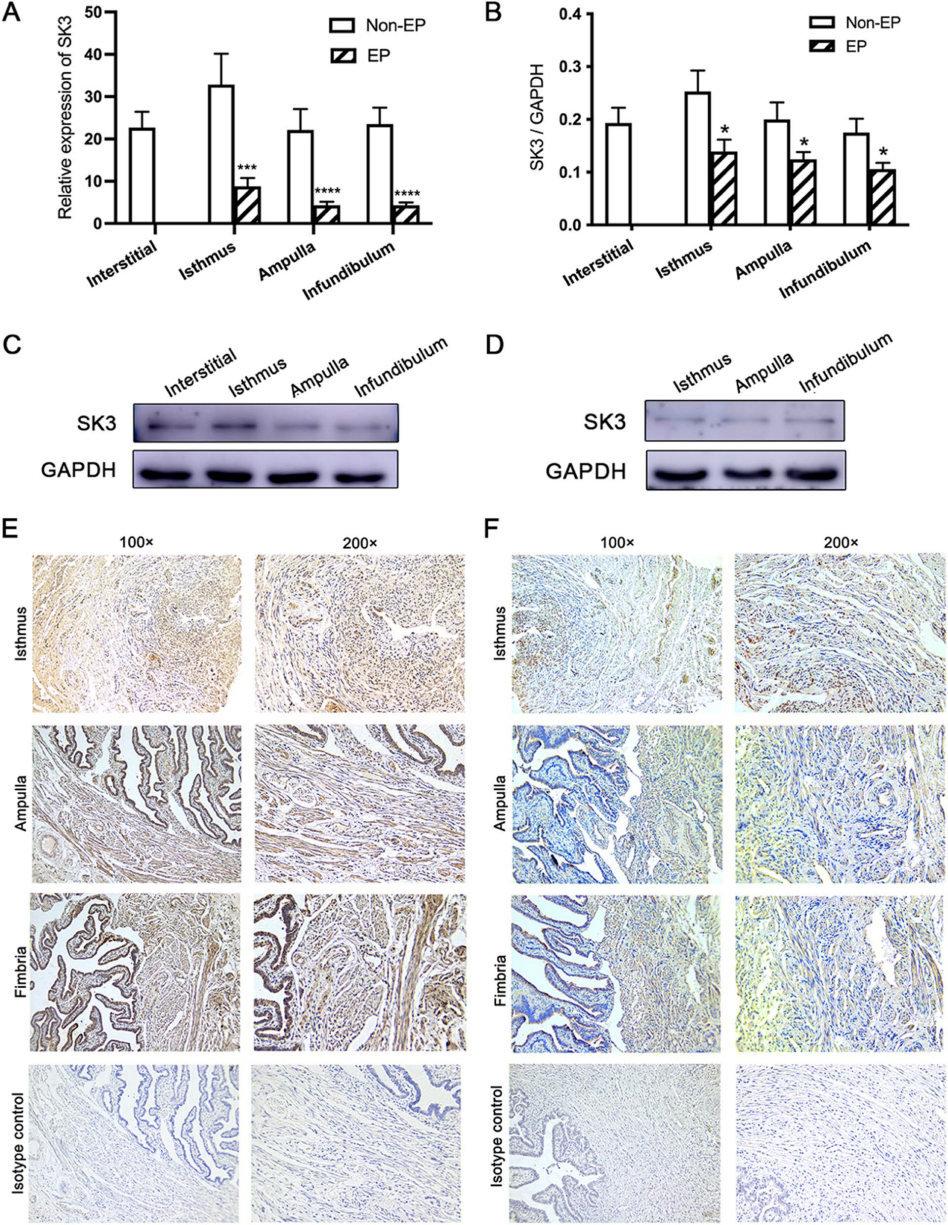
The expression of SK3 in the hFTSM. **A** The mRNA level of SK3 in the Non-EP and EP group and the difference in SK3 mRNA level between two groups. **B** Gray intensity analysis and comparison of the protein levels of SK3 in the two groups. **C** The protein level of SK3 in the Non-EP group. **D** The protein level of SK3 in the EP group. SK3 protein is expressed in the tubal endometrium and smooth muscle tissue of the Non-EP (**E**) and EP (**F**) groups. sm, smooth muscle; tl, tubal lumen; te, tubal epithelium. *, *P*<0.05; ***, *P*<0.001; ****, *P*<0.0001. (significant difference detected between Non-EP and EP groups)

To explore the mRNA and protein levels of SK3 in the hFTSM, we performed qRT-PCR, western blot, and immunohistochemistry analyses. The mRNA level of SK3 did not differ between different portions of the fallopian tube within each group (Fig. 2A, B), and the levels of SK3 in the isthmus, ampulla, and infundibulum from the Non-EP group were significantly higher than those from the EP group (Fig. 2C). The protein levels of SK3 in different parts of the fallopian tubes from the two groups were basically in line with the mRNA levels (Fig. 2D-F). Immunohistochemical staining showed that SK3 is expressed in the endosalpinx and hFTSM in both groups (Fig. 2G-H). Similar to the results of PCR and western blot, the expression of SK3 in hFTSM of different portions is much more in non-EP group than EP group (Fig. 2G-H).

### The effect of apamin on progesterone-induced inhibition of tubal contraction

**Fig. 3:**
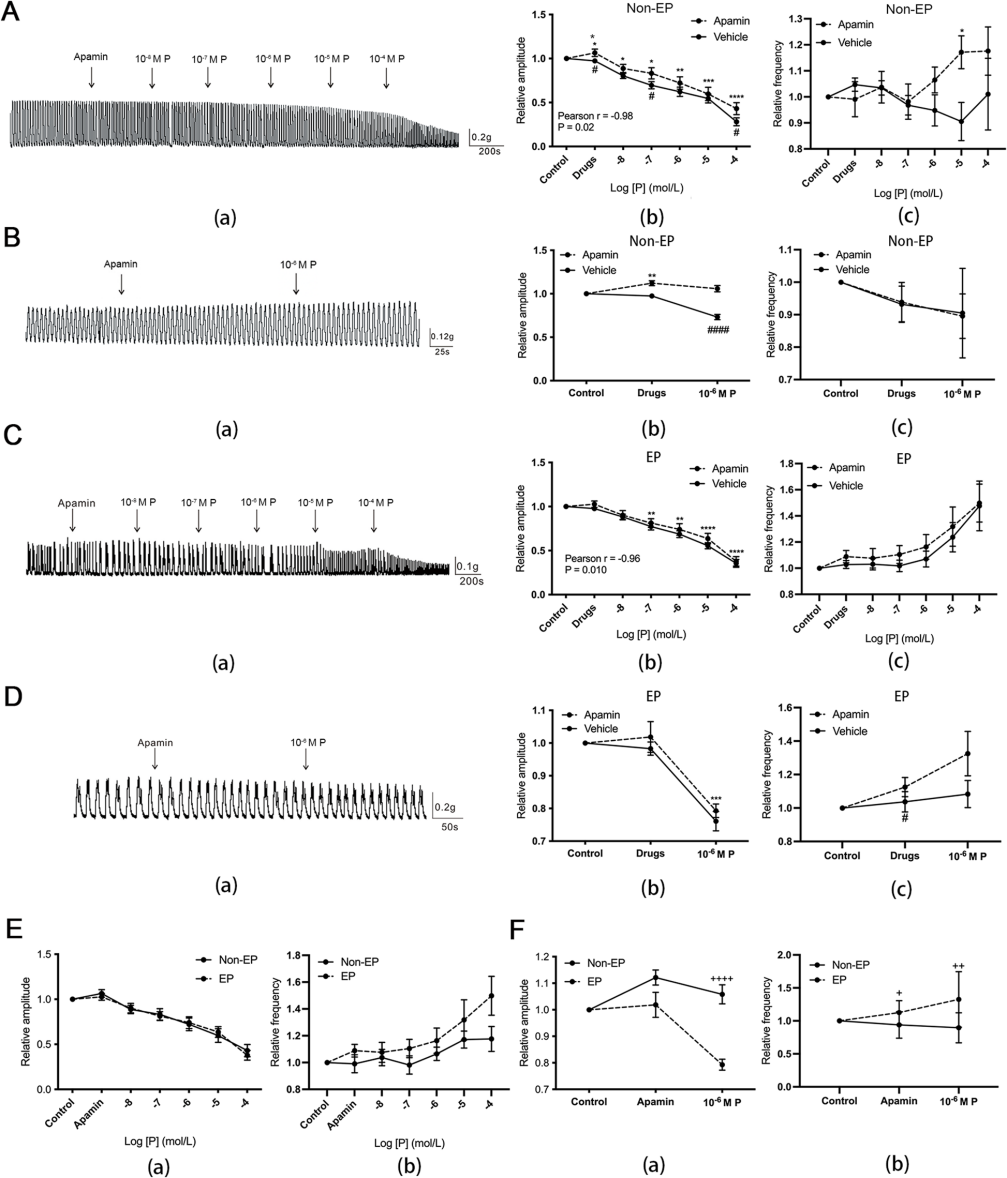
Effect of apamin on progesterone-induced inhibition of the spontaneous contraction of the hFTSM. **A**-**B** The effect of increasing concentrations of progesterone (**A**) and 10^− 6^ M progesterone (**B**) on the amplitude (**a**) and frequency (**b**) of spontaneous contraction of hFTSM from the Non-EP group pretreated with apamin. **C**-**D** The effect of increasing concentrations of progesterone (**C**) and 10^− 6^ M progesterone (**D**) on the amplitude (**a**) and frequency (**b**) of spontaneous contraction of the hFTSM from the EP group pretreated with apamin. **E**-**F** Comparison of the effects of increasing concentrations of progesterone € and 10^− 6^ M progesterone (**F**) on the amplitude (**a**) and frequency (**b**) of spontaneous contraction of the hFTSM pretreated with apamin between the two groups. *, *P*<0.05; **, *P*<0.01; ***, *P*<0.001; ****, *P*<0.0001 (significant difference detected within a group as compared with the baseline amplitude/frequency. #, *P*<0.05; ##, *P*<0.01; ###, *P*<0.001; ####, *P*<0.0001 (significant difference detected between apamin-pretreated and vehicle-pretreated group). ++, *P*<0.01; ++++, *P*<0.0001 (significant difference detected between Non-EP and EP groups)

In the control apamin-pretreated (AP) subgroup, the amplitude increased significantly in the presence of apamin, a SK channel blocker, and it decreased significantly and dose-dependently with the subsequent addition of progesterone (Fig. 3A-B: (a)-(b); Pearson R = -0.98, *P* = 0.02). However, the amplitude in the control AP subgroup remained unchanged compared with the baseline when 10^− 6^ M progesterone was added (Fig. 3B(b)). The inhibition of amplitude by the addition of a series of increasing concentrations of progesterone (10^− 7^–10^− 4^ M) and 10^− 6^ M progesterone alone was weaker in the AP subgroup compared with the control vehicle-pretreated (VP) subgroup (Fig. 3A-B: (a)-(b)). In the EP group, the amplitude was significantly reduced by the addition of increasing concentrations of progesterone from 10^− 7^ to10^− 4^ M or 10^− 6^ M progesterone alone; however, no difference in the reduction in amplitude was observed in the AP subgroup (Fig. 3C-D: (a)-(b)). Apart from the increased frequency in the control AP subgroup under 10^− 5^ M progesterone (Fig. 3A (c)), the frequencies were fundamentally invariant with increasing concentrations of progesterone in each subgroup.

The effect of apamin on amplitude and frequency in response to increasing concentrations of progesterone were similar between the two groups (Fig. 3E), but the amplitude in the presence of 10^− 6^ M progesterone was much greater in the Non-EP AP subgroup than in the EP AP subgroup (Fig. 3F (a)), while the frequency was lower in Non-EP AP subgroup (Fig. 3F (b)). In short, apamin can help to alleviate the inhibition of progesterone in contractile amplitude to some degree in two groups.

### The effect of TEA on progesterone-induced inhibition of tubal contraction

**Fig. 4:**
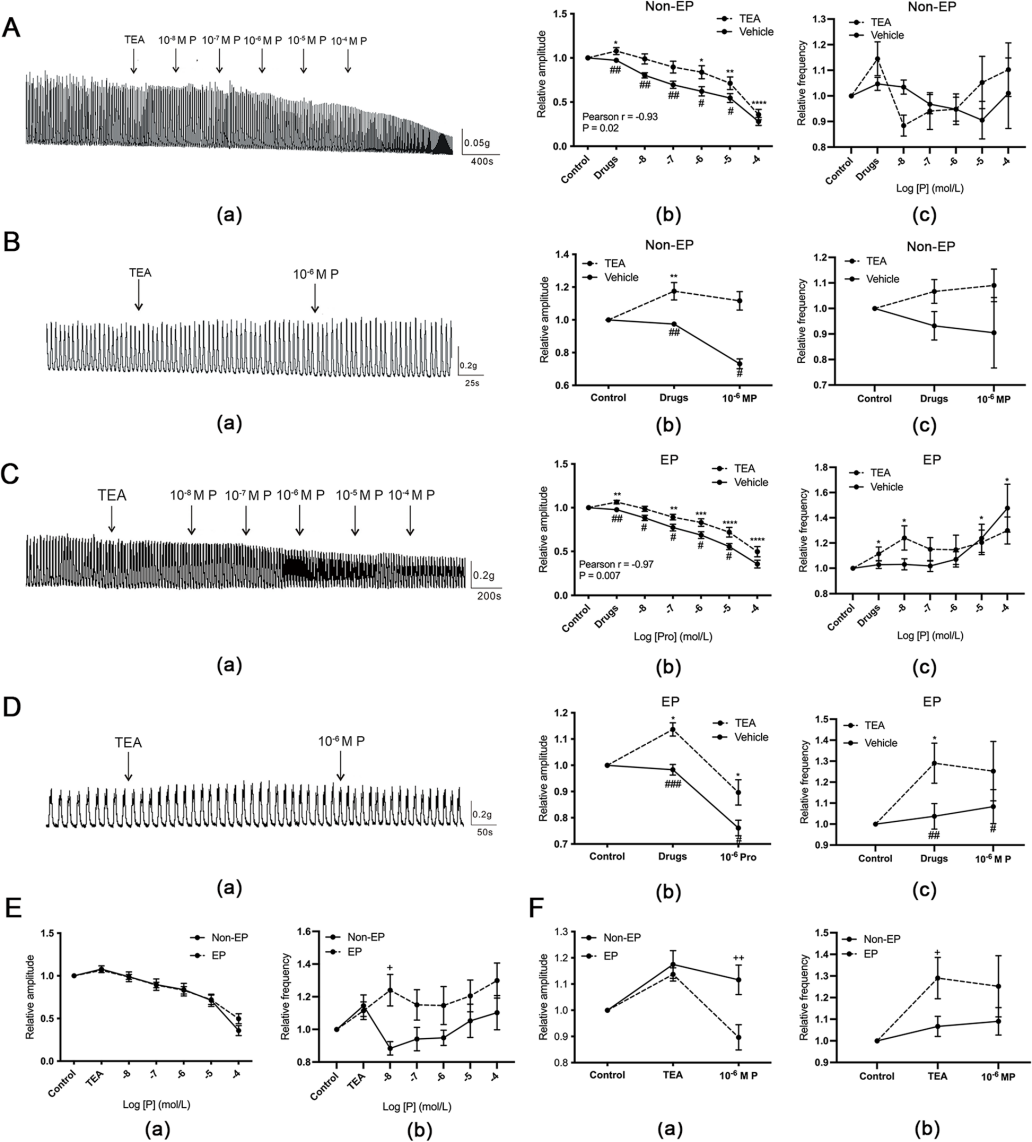
Effect of TEA on progesterone-induced inhibition of spontaneous contraction of the hFTSM. **A**-**B** The effect of increasing concentrations of progesterone (**A**) and 10^− 6^ M progesterone (**B**) on the amplitude (**a**) and frequency (**b**) of spontaneous contraction of the hFTSM from the Non-EP group pretreated with TEA. **C**-**D** The effect of increasing concentrations of progesterone (**C**) and 10^− 6^ M progesterone (**D**) on the amplitude (**a**) and frequency (**b**) of spontaneous contraction of the hFTSM from the EP group pretreated with TEA. **E**-**F** Comparison of the effects of increasing concentrations of progesterone (**E**) and 10^− 6^ M progesterone (**F**) on the amplitude (**a**) and frequency (**b**) of spontaneous contraction of the hFTSM pretreated with TEA between the two groups. *, *P*<0.05; **, *P*<0.01; ***, *P*<0.001; ****, *P*<0.0001 (significant difference detected within a group as compared with the baseline amplitude/frequency). #, *P*<0.05; ##, *P*<0.01; ###, *P*<0.001 (significant difference detected between the apamin-pretreated and vehicle-pretreated groups). +, *P*<0.05; ++, *P*<0.01 (significant difference detected between the Non-EP and EP groups)

We incubated muscle strips in TEA and examined its effects on progesterone-induced inhibition of tubal contraction. The results showed that in both the Non-EP and EP groups, the amplitude increased in the presence of TEA (Fig. 4A-D:(a)-(b)). In the control TEA-pretreated (TP) subgroup, the amplitude remained unchanged compared with the baseline when increasing concentrations of progesterone from 10^− 8^ M and 10^− 7^ M were added and when 10^− 6^ M progesterone was added alone (Fig. 4A-B: (b)). However, the amplitude was dose-dependently weakened when progesterone was added at concentrations higher than 10^− 7^ M (Fig. 4A (b); Pearson *R* = -0.93, *P* = 0.02). The inhibition induced by successively increasing concentrations of progesterone ranging from 10^− 8^–10^− 5^ M or a single application of 10^− 6^ M progesterone was partly reversed by TEA (Fig. 4A-B : (b)).

Similar to the Non-EP group, the amplitude in the EP group gradually decreased at progesterone concentrations 10^− 7^ M and higher (Fig. 4C (b); Pearson R = -0.97, *p* = 0.007). What was different from the Non-EP group was that the decrease in amplitude was significant with the addition of 10^− 6^ M progesterone alone (Fig. 4D (b)). Also, the inhibition under increasing concentrations of progesterone from 10^− 8^–10^− 5^ M or 10^− 6^ M progesterone was counteracted by TEA (Fig. 4C-D: (b)). Pretreatment with TEA and subsequent treatment with progesterone had no effect on the frequency in the Non-EP group (Fig. 4A-B : (c)), whereas TEA increased the frequency in the EP group (Fig. 4C-D : (c)).

With respect to the effect of increasing concentrations of progesterone on amplitude following pretreatment with TEA, there was no difference between the Non-EP and EP groups (Fig. 4E (a)), but the amplitude in the EP TP subgroup was significantly smaller than that in the Non-EP TP subgroup when 10^− 6^ M progesterone was added (Fig. 4J (a)). The frequency increased in the EP group upon pretreatment with TEA (Fig. 4J (b)). In summary, TEA can increase the contractile amplitude and counteract the inhibitory effect of progesterone in contractile amplitude in two groups.

### The existence of SK3-expressing PDGFRα (+) cells in the hFTSM

**Fig. 5:**
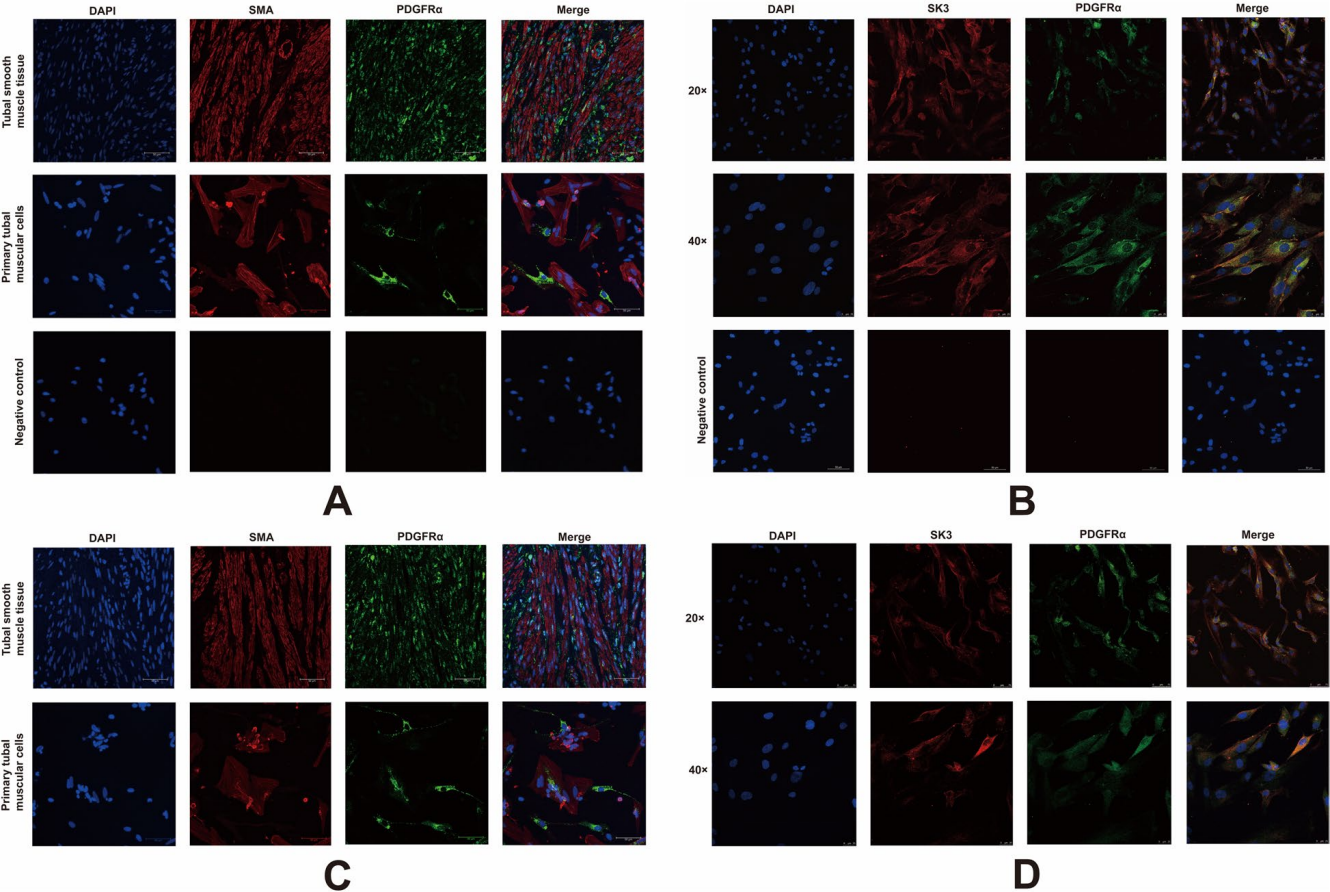
The expression of SK3 in PDGFRα (+) cells. **A, C** Localization of PDGFRα (+) cells in the human fallopian tube muscular layer in the Non-EP group (A) and EP group (**C**). **B**, **D** SK3 expression in PDGFRα (+)primary smooth muscle cells from the Non-EP group (**B**) and EP group (**D**)

To explore the existence of SK3-expressing PDGFRα (+) cells in the hFTSM, immunohistochemical and immunocytochemical staining were performed. Spindle-shaped or stellate-shaped PDGFRα (+) cells were scattered among the SMCs in hFTSM sections from the Non-EP group (Fig. 5A) and EP group (Fig. 5C). SK3 was abundantly expressed in PDGFRα (+) cells in both the Non-EP group (Fig. 5B) and EP group (Fig. 5D), and it was also weakly expressed in other cells.

## Discussion

EC serves as an important back-up method for unprotected intercourse or unexpected failure of regular contraceptives; thus, it is a critical part of family planning services [30]. The elevated risk of EP following failure of EC with LNG or combined oral contraceptives prompted us to look at the effect of progesterone on the fallopian tube [6, 31]. The attenuation of hFTSM contraction by progesterone has been widely recognized, and this effect might be of significance in the pathogenesis of EP following EC failure and the transportation of pre-embryos to the uterine cavity [9]. It has been reported that endothelin and progesterone receptors (PRs) including PRA, PRB, and membrane PR are expressed and involved in progesterone-induced effects on fallopian tubes [9, 32–34]. However, there have been few studies of the role of ion channels in the rapid effects of progesterone on hFTSM contraction. To our knowledge, this is the first study investigating the expression and function of SK3 in fallopian tube contraction in EP and non-EP patients.

Previous studies concluded that the amplitude of hFTSM contraction is similar at different phases of normal menstrual cycles, whereas the frequency is significantly higher during the periovulatory period [35]. Lower frequency of tubal contraction was observed in early pregnancy, the latter part of the luteal phase, and upon treatment with combined oral contraceptives [35]. Moreover, the frequency is higher in the tubal circular layer than in the longitudinal layer during the ovulatory phase [36]. As previous studies reported, a progesterone-induced decrease in contraction amplitude was observed in both the EP and Non-EP groups. The results of the present study suggest that there is a significantly decreased baseline amplitude and frequency in the EP group compared with the Non-EP group, and this may be attributed to elevated serum levels of progesterone and human chorionic gonadotropin and tubal damage (edema, hyperemia, and myofiber fracture) caused by ectopic trophoblasts in the EP group [9, 37]. The two groups share a basically similar contractile pattern in the presence of progesterone except for the interesting observation that in EP group tubal contraction progressed to a more rapid flutter with the addition of 10^− 5^ M progesterone.

There has been no study examining the expression of SK3 in the fallopian tubes from humans or other species so far. The expression of SK3 varies in different menstrual phases and during pregnancy, and its relationship with estrogen and progesterone is complicated [24, 38–41]. Several studies have reported a significant decrease in SK3 expression in the myometrium from pregnant women and mice compared with non-pregnant tissue [24, 38, 39]. This phenomenon may be ascribed to hypertrophy and a larger cell volume in pregnant myometrium or to transcriptional regulation by hormones [39]. Apart from above factors, the age of patients is also an important factor due to the significant difference in age between EP and Non-EP groups [39]. Besides, significant more gravities and parities in Non-EP group may be attributed to the older age of Non-EP patients. The higher number of patients with recurrent EP in EP group suggests their impaired tubal function and high risk in EP. Although there was no significant difference in weight between two groups, whether Body Mass Index can influence the tubal contraction remains unknown for the lack of patients’ height in our study.

Apamin is the most widely known SK channel blocker; it blocks the outflow of K^+^ via the SK1, SK2, and SK3 channels and relieves the cell from prolonged hyperpolarization, thus causing enhanced contraction, whereas TEA is a non-specific KCa blocker [42]. Anderson L et al. demonstrated that none of the potassium channels (including KATP, BKCa, IKCa, and SKCa) are involved in progesterone-induced inhibition in the myometrium from pregnant women [43]. Here we revealed that blocking KCa with TEA leads to an increased contraction amplitude and almost complete attenuation of progesterone-induced inhibition in the two groups. In contrast, apamin only increased the amplitude and partially counteracted progesterone-induced inhibition in the Non-EP group, which may be related to the higher expression of SK3 and consequent increased sensitivity to apamin in Non-EP women. Moreover, in the presence of apamin, the similar change in contraction following increasing concentration of progesterone in two groups, which was not corresponding to the different expression of SK3 between two groups, may attributed to progesterone receptors or other ion channels potentially involved in the progesterone-induced inhibition. The results indicate that SK3 plays an important role in the inhibition of spontaneous tubal contraction induced by progesterone, and other subtypes of KCa channels may also be involved in this process.

Multiple kinds of cells are involved in the intricate motor activity of the fallopian tube, including SMCs, intramural neurons, and interstitial cells, which act like pacemakers (interstitial cells of Cajal [ICC]) [28] Contrary to the ICC, PDGFRα (+) cells are a population of interstitial cells that are closely associated with nerve fibers and form gap junctions with SMCs, and they mediate inhibitory neurotransmission in many organs [28]. PDGFRα (+) cells have been demonstrated to be distributed along the fallopian tube in monkeys, and they mainly exist in the serosa of the ampulla and isthmus and are scattered in the muscular layer [28]. Apart from detrusor, gastric fundus, and colon from murine, SK3-expressing PDGFRα (+) cells are also found in the hFTSM as shown in the present study [24, 44, 45]. Therefore, we hypothesize that progesterone may play an inhibitory role in fallopian tubal contraction through SK3 in PDGFRα (+) cells. And the inhibitory signal may be passed to surrounding SMCs from PDGFRα (+) cells, thus leading to muscle relaxation.

In summary, we found that the baseline amplitude and frequency of fallopian tube contraction are both statistically lower in the EP group compared with the Non-EP group. The expression levels of SK3 in different portions of fallopian tubes from the Non-EP group were significantly higher than those from the EP group. Progesterone plays an inhibitory role in tubal contraction, mainly by affecting amplitude, in the two groups, and SK3 as well as other KCas may be involved. Finally, SK3-expressing PDGFRα (+) cells were demonstrated to exist in the human fallopian tube.

There are still limitations to the present study. Failing to separate and analyze different muscular layers (circular and longitudinal layer) makes the results less precise owing to the different gene expression and contractile patterns of these layers. Moreover, considering the asynchronous activity of different portions of the fallopian tube, the responses of different regions to progesterone and KCa channel blockers should be studied. In consideration of the difference in hormone levels between EP and non-EP group, it is necessary to detect the complicated correlation between progesterone or estradiol level and the expression of SK3 or other calcium-activated potassium channels in primary tubal smooth muscle cells in further studies. The deeper underlying mechanisms, i.e., the effect of progesterone on ion flow and membrane potential and the way it interacts with SK3 and PDGFRα (+) cells, needs to be further explored.

## Supplementary Information

**Additional file 1: Supplementary Table 1**. Antibodies used for western blotting (WB), immunohistochemisctry (IHC) and immunocytochemistry (ICC).

**Additional file 2: Supplementary Table 2**. Drugs and chemicals used in the isometric tension experiment.

## Data Availability

The data and materials are available from the corresponding author on reasonable requests.
