# Supplementary material for: The role of SK3 in progesterone-induced inhibition of human fallopian tubal contraction
Source: Reprod Biol Endocrinol. 2022 Apr 29;20:73. doi: 10.1186/s12958-022-00932-3 (PMC9052544; doi:10.1186/s12958-022-00932-3)
Supplement: Supplementary file 1 — Additional file 1: Supplementary Table 1. Antibodies used for western blotting (WB), immunohistochemisctry (IHC) and immunocytochemistry (ICC). [file 12958_2022_932_MOESM1_ESM.docx]

Supplementary Table 1. Antibodies used for western blotting (WB), immunohistochemisctry (IHC) and immunocytochemistry (ICC).

|  | Antibodies | Dilution/  Concentration | Source | Identifier |
| --- | --- | --- | --- | --- |
| Primary antibodies for IHC | Rabbit anti-SK3 | 1:100 | Abcam | Cat# ab220864;RRID:NA |
|  | Mouse anti-α-SMA | 1:1000 | Abcam | Cat# ab7817;RRID:AB_262054 |
|  | PDGFRα (D1E1E) XP^®^ Rabbit mAb | 1:1000 | Cell Signaling Technology | Cat# 3174S;RRID: AB_2162345 |
| Secondary antibodies for IHC | HRP-labeled anti-rabbit IgG | 1:200 | Cell Signaling Technology | Cat# 7074;RRID: AB_2099233 |
|  | Alexa Fluor 594-conjugated anti-Mouse IgG | 1:200 | Thermo Fisher | Cat# A-11005;RRID: AB_2534073 |
|  | Alexa Fluor 488-conjugated anti-Rabbit IgG | 1:500 | Thermo Fisher | Cat# A-11008;RRID:AB_143165 |
| Primary antibodies for WB | rabbit anti-SK3 antibody | 2μg/ml | Abcam | Cat# ab28631;RRID: AB_775888 |
|  | GAPDH (D16H11) XP^®^ Rabbit mAb | 1:1000 | Cell Signaling Technology | Cat# 5174;RRID:AB_10622025 |
| Secondary antibodies for WB | HRP-labeled anti-rabbit IgG | 1:200 | Cell Signaling Technology | Cat# 7074;RRID:AB_2099233 |
| Primary antibodies for ICC | Rabbit anti-α-SMA | 1:200 | Proteintech | Cat# 55135-1-AP;RRID:AB_10949628 |
|  | Rabbit anti-SK3 | 1:20 | Proteintech | Cat# 17118-1-AP;RRID:AB_2878358 |
|  | Mouse anti-PDGFRα | 1:100 | Novus Biologicals | Cat# NBP2-52528;RRID:NA |
| Primary antibodies for ICC | Alexa Fluor 594-conjugated anti-Rabbit IgG | 1:500 | Proteintech | Cat# SA00006-4;RRID:AB_2756337 |
|  | Alexa Fluor 488-conjugated anti-Mouse IgG | 1:500 | Thermo Fisher | Cat# A-11001;RRID:AB_2534069 |
|  | Alexa Fluor 568-conjugated anti-Rabbit IgG | 1:500 | Thermo Fisher | Cat# A-10042;RRID:AB_2534017 |
