# Supplementary material for: The role of SK3 in progesterone-induced inhibition of human fallopian tubal contraction
Source: Reprod Biol Endocrinol. 2022 Apr 29;20:73. doi: 10.1186/s12958-022-00932-3 (PMC9052544; doi:10.1186/s12958-022-00932-3)
Supplement: Supplementary file 2 — Additional file 2: Supplementary Table 2. Drugs and chemicals used in the isometric tension experiment. [file 12958_2022_932_MOESM2_ESM.docx]

| Drugs/Chemicals |  | Components | Source | Identifier |
| --- | --- | --- | --- | --- |
| Krebs solution |  | NaCl (137.4. mmol/L) | Sangon Biotech | Cat# A100241 |
|  |  | KCl (5.9 mmol/L) |  | Cat# A100395 |
|  |  | NaHCO_3_ (15.5 mmol/L) |  | Cat# A100865 |
|  |  | KH_2_PO_4_ (1.2 mmol/L) |  | Cat# A100781 |
|  |  | MgSO_4_ (1.2 mmol/L) |  | Cat# A601988 |
|  |  | D-glucose (11.5 mmol/L) |  | Cat# A501991 |
|  |  | CaCl_2_ (2.5mmol/L) * |  |  |
| TEA |  | 5 mmol/L | TOCRIS Bioscience | Cat# 3068/50 |
| Apamin |  | 300 nmol/L | TOCRIS Bioscience | Cat# 1652/1 |
| Progesterone |  |  | Sigma Aldrich | P0130 |

Supplementary Table 2. Drugs and chemicals used in the isometric tension experiment
